# Effect of a Comprehensive Rehabilitation Program for Community Women with Urinary Incontinence: A Retrospect Cohort Study

**DOI:** 10.3390/healthcare9121686

**Published:** 2021-12-06

**Authors:** Sen-Ju Yang, Yi-Ting Liu, Su-Shun Lo, Chih-Chun Tsai, Po-Jung Pan

**Affiliations:** 1 Department of Physical Medicine & Rehabilitation, National Yang Ming Chiao Tung University Hospital, Yilan 260002, Taiwan; 15103@ymuh.ym.edu.tw (S.-J.Y.); 15063@ymuh.ym.edu.tw (Y.-T.L.); 2 School of Medicine, National Yang Ming Chiao Tung University, Taipei 112304, Taiwan; sslo@ymuh.ym.edu.tw; 3 Department of Mathematics, Tamkang University, New Taipei City 251301, Taiwan; chihchuntsai@mail.tku.edu.tw

**Keywords:** biofeedback, pelvic floor muscle training, quality of life, urinary incontinence

## Abstract

Urinary incontinence (UI) is a common problem affecting older adult women globally, but studies regarding combined treatments for all types of UI are still lacking. Here we evaluate the efficacy of a comprehensive rehabilitation program for women with UI. A comprehensive rehabilitation program was introduced that combines pelvic floor muscle (PFM) exercises, functional electrical stimulation, and timely biofeedback during the training process. Data of patients with stress (SUI), urgency (UUI), or mixed (MUI) urinary incontinence who participated in this program between 2016 and 2019 were reviewed retrospectively. Seventy-three subjects (mean age 59.2 ± 12.7 years) were enrolled. After 12 weeks of rehabilitation, vaginal pressure and control accuracy increased in all groups. PFM maximum recruitment increased significantly at week 12 in SUI and UUI, but not in MUI. At week 6, only the SUI group had achieved significant improvements in vaginal pressure, PFM maximum recruitment and control accuracy. The Short-form Urogenital Distress Inventory (UDI-6) and Incontinence Impact Questionnaire-7 (IIQ-7) scores declined dramatically after the program started, and significant improvements were maintained to week 48. The comprehensive rehabilitation program is effective and decreases urinary leakage episodes and improves quality of life in women with UI, especially SUI.

## 1. Introduction

Urinary incontinence is a common problem among older adult women globally. The International Continence Society defines female urinary incontinence as involuntary leakage of urine that is detectable objectively and may cause social or hygienic distress [1]. According to data adopted by the 6th International Consultation on Incontinence, urinary incontinence accounts for 4% to 8% of the world’s population and 383 million people were estimated to be affected in 2013 [2]. The prevalence of urinary incontinence among women in the community is about 15–30% [3], and studies have found that urinary incontinence increases with age, mostly due to age-related changes in the urinary system, including organ mucosal atrophy or changes in bladder volume and hypermobility of the bladder neck [4].

Based on clinical interviews and self-evaluation questions, urinary incontinence can be divided into three different types: stress urinary incontinence (SUI), which is about 51% of total urinary incontinence; urgency urinary incontinence (UUI), representing about 10%; and mixed urinary incontinence (MUI), accounting for about 39% [4]. SUI occurs when the abdominal pressure increases (during physical activities such as coughing, sneezing, laughing, running and jumping, heavy-lifting, or going down stairs) without perception of the desire to void; UUI indicates urine leakage occurring before reaching the toilet when there is an urgency to void, which is caused by bladder muscle spasm or overactive bladder, and the underlying mechanisms cannot be identified in most cases; and MUI is a combination of SUI and UUI [4,5].

The first choice of treatment for urinary incontinence is typically conservative in patients who fear or reject surgery, or in patients with severe cardiovascular disease, stroke, diabetes, or other chronic diseases who are not suitable for surgery. Conservative treatments for urinary incontinence include biofeedback training, electrical stimulation, pelvic floor muscle (PFM) exercise, urination training, medication, and magnetic stimulation [6]. Many women do not understand how PFM work, and, in fact, about 30–50% of women don’t know how to actively contract the PFM. Effective PFM training is closely associated with whether the patient can properly contract PFM. Generally, patients are advised to try avoiding contracting other muscles related to the pelvic floor at the same time as they are contracting PFM. Many women use other muscles such as the gluteal muscles, thigh adductors and abdominal muscles for compensatory contraction, and holding the breath or deep inhalation can also cause the PFM to move downwards. When these muscles contract, some pressure is placed on the vagina, but little effect on the urethra. Patients often contract these compensatory muscles and mistakenly believe that this meets the requirements of PFM contraction, so the training results are often not satisfactory [7,8,9].

Biofeedback therapy places pressure gauges or recording electrodes in the vagina, converting the changes in vaginal pressure or electromyography (EMG) signals into a graphic display in time. This method also can detect the EMG of the abdominal muscles to remind the trainees to avoid using compensatory muscles to exert force. The therapist instructs the trainee to perform the correct PFM contraction–relaxation through auditory and visual feedback, thereby strengthening the PFM and coordination control, improving the functionality of the pelvic muscles, enhancing PFM support for the pelvic organs and finally improving urinary incontinence [10]. Intravaginal electrical stimulation (IES) can be used to treat stress or urgency urinary incontinence [11]. Stimulating the nerves by placing electrodes with different electrical stimulation frequencies in the vagina reduces urine leakage, frequent urination, and nocturia. IES therapy activates PFM, reduces compensatory muscle contraction, improves urination, relieves bladder overactivity, and increases bladder capacity. Exercise therapy mainly strengthens PFM and core muscles and maintains the therapeutic outcome for a longer period [12].

Clinically, patients with urinary incontinence manifest different symptoms and different rates of disease progression. Therefore, a comprehensive rehabilitation program uses different combinations of conservative treatments based on patients’ actual clinical conditions, and different treatment models are designed to satisfy individual patients’ needs. Patients with more severe conditions who cannot perform active PFM training are recommended to be treated with electrical stimulation first. Patients with milder conditions or those showing improvement during the treatment process will usually be treated with the biofeedback therapy to provide accurate training and to increase endurance. During the treatment process, exercise therapy will be integrated for all patients to strengthen the PFM and help develop the habit of self-training at home. Previous studies have mostly targeted SUI [13] or single treatment intervention [14] to evaluate the outcomes. The aim of this study is to investigate the efficacy of a comprehensive rehabilitation program combining biofeedback therapy with IES and exercise therapy for women with urinary incontinence.

## 2. Materials and Methods

### 2.1. Study Design and Subjects

This retrospective cohort study analyzed the data of consecutive female patients with urinary incontinence who participated in a comprehensive rehabilitation program at National Yang Ming University Hospital between 1 July 2016 and 31 December 2019. The program was conducted by a multidisciplinary team composed of members of the Department of Obstetrics and Gynecology, Department of Urology, and Department of Physical Medicine and Rehabilitation of National Yang Ming University. Initially, the obstetricians and gynecologists determined the eligible subjects; they diagnosed and distinguished SUI, UUI, and MUI based on the patients’ main complaints or symptoms, urodynamic testing results, and clinical interview. Inclusion criteria were women with SUI, UUI, or MUI. Women with neurogenic bladder, gynecological, or urinary tract infections, infectious diseases, and pelvic organ prolapse greater than Grade III were excluded. Based on urinary incontinence types, the included subjects were grouped into SUI, UUI, and MUI groups. Demographic, anthropometric, and clinical data were collected, and the Short-form Urogenital Distress Inventory (UDI-6) and Short-form Incontinence Impact Questionnaire-7 (IIQ-7) were administered to the included subjects [15]. Urodynamic examination was performed by a urologist for all included patients. This study mainly relied on management provided by the Department of Physical Medicine and Rehabilitation. Treatments were prescribed afterwards and were performed by the same two physical therapists.

### 2.2. Ethical Considerations

The protocol for this study was reviewed and approved by the Institutional Review Board (IRB) of National Yang Ming University Hospital (IRB 2021A003). All the participants were informed by the case manager of the entire treatment program in detail, and they consented and assured to follow the protocol during the study period. At the time of program participation, all included patients provided signed informed consent for their data to be used for later evaluation and publication.

### 2.3. Evaluation and Main Measures

**Figure 1:**
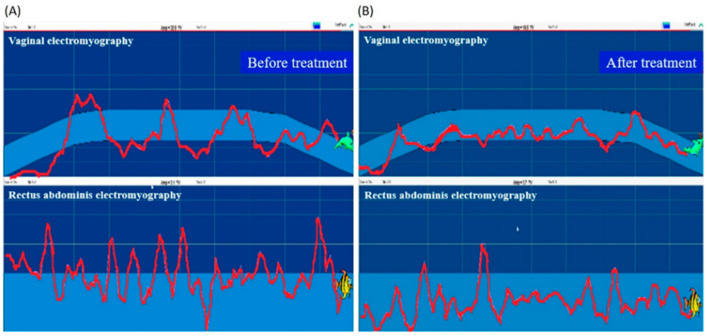
An illustration of the biofeedback mode during the treatment process. (**A**) Before the rehabilitation program. (**B**) After the rehabilitation program.

At each patient’s first visit, the physical therapist collected the demographic, anthropometric, and gynecologic information, and assessed the PFM and the grading of pelvic organ prolapse, using the PERFECT scheme [16]. Pelvic examination was performed; each subject lay down on her back with her hips and knees bent at 45 degrees and underwent transvaginal digital palpation. The subjects were guided to complete the assessment, and measures of muscle power (P), endurance (E), repetitions (R), fast contractions (F), and every contraction timed (ECT) were recorded [16]. The level of pelvic organ prolapse and the presence of activation of compensatory muscles, including abdominal muscles, gluteal muscles and hip adductors, were recorded. The built-in manometry and electromyography of the biofeedback device Urostym^®^ (Laborie Co., Mississauga, ON, Canada) were used to detect PMF contraction and activity. Vaginal manometry connected to a sensor was used to measure vaginal resting pressure (in mmHg) or pressure rise during PFM contraction [17]. PFM maximum recruitment (uV) and control accuracy (%) were recorded. Control accuracy was arbitrarily defined as “the percentage of the time duration during which the patient can control her PFM contraction (illustrated as the trajectory of vaginal electromyography activity) relative to a time frame with a pre-designed difficulty level” according to the manufacturer’s instructions. Data of PFM maximum recruitment were converted from physiological and electrical signals of muscles during activity or at rest, which was measured five times and the average of the highest three values was taken as the PFM maximum recruitment value. Control accuracy referred to the percentage of cases that completed the Urostym^®^ protocol at preset intervals, which was specifically designed for recording and evaluating the control force of PFM with a vaginal probe. This value was also measured five times and the average of the highest three values was taken as the PFM control force. The pressure probe was placed at the depth of three to four centimeters of the vaginal opening. During the measurement, the pressure signal of vaginal contraction was converted into a graphic display on the screen. Based on this visual assessment, the physical therapist guided the patient to correctly contract the PFM to achieve a satisfactory therapeutic effect. This process was also combined with functional electrical stimulation. An electrical stimulation probe with appropriate intensity (0–70 mA), wavelength (200–500 us), and pulse (1–3 ms) was placed in the vagina, and electrical stimulation was applied via the metal ring of the probe to induce activation of the PFM. The game mode of the biofeedback device was also used during the process to promote the PFM exercise, increase the fun of treatment, and enhance the correctness and performance skills of the PFM exercise (Figure 1 comparing [B] with [A], shows that the vaginal contraction force recorded as electromyography activity is better controlled within the game interval, which indicates better control accuracy; the rectus abdominis electromyography activity is also lower, which indicates less abdominal wall compensatory contraction).

### 2.4. Treatment Plan

Participating patients were required to receive a 12-week comprehensive rehabilitation program, twice a week (one instrument therapy, one exercise training), with instrument therapy for 15 to 20 min each time, and exercise training for 30 min each time. The exercise training included PFM contractions (Kegel exercises), core muscle strengthening (sit-ups, planks, and side planks), and lower limb strength training. The physical therapists guided the patients to contract the PFM with the following verbal commands similar to those reported by Reis et al. [18]: (i) contract the PFM as if you are holding the urine; (ii) make an upwards and inwards muscle movement; (iii) inhale when your muscles are relaxed and exhale when your muscles are contracting; and (iv) avoid contracting the abdomen, glutes, or leg muscles while contracting the pelvic floor muscles. Individual exercise training programs were designed according to the needs of each subject. Adherence of the patient to the protocol was checked by consulting. Vaginal pressure, PFM maximum recruitment, and control accuracy were evaluated at baseline (before treatment), 6 weeks, and 12 weeks after treatment. UDI-6 and IIQ-7 questionnaires were evaluated at baseline and 6, 12, and 24 weeks after treatment. At baseline and for 3 consecutive days after treatment, the bladder diary was self-recorded by the patient for daily water intake, daily frequency of urination, daily urine output, daily frequency of incontinence episodes, daily urinary urgency episodes, and daily nocturia, to assess the urinary incontinence [19].

### 2.5. Statistical Analysis

The collected data were coded to establish a computer file and analyzed using SPSS 22.0 software (IBM Corp., Armonk, NY, USA). Demographic, anthropometric, and gynecologic data, including the number of patients, age, percentage of menopause patients, weight, height, body mass index (BMI), number of births, percentage of parity >2, and percentage of gynecological surgery history were reported with descriptive statistics. Categorical data are presented as numbers (%) and continuous data are presented as mean ± standard deviation (mean ± SD). Normality checks were carried out on the residuals which were approximately normally distributed. Paired *t*-tests were used to compare vaginal pressure, PFM maximum recruitment, and control accuracy at baseline and at weeks 6 and 12 after intervention. UDI-6 scores and IIQ-7 scores within SUI, UUI, and MUI groups over time were compared using paired *t*-test or repeated measured ANOVA with appropriate post hoc test. Differences between the menopause group and the pre-menopause group in control accuracy, and differences between parity groups in vaginal pressure at baseline and weeks 6 and 12 after intervention were compared using two-way mixed ANOVA with post hoc test (least significant difference). Logistic regression analysis using forward stepwise variable selection method for variables including age, BMI, menopause, total number of births, gynecological surgery, SUI, UUI, and MUI was performed among the patients who achieved dryness after the rehabilitation program. *p* < 0.05 indicated statistical significance in all analyses.

## 3. Results

Seventy-three subjects were recruited. The demographic, anthropometric, and gynecologic characteristics of all subjects and of the SUI, UUI, and MUI subgroups are shown in Table 1. All the included women had given birth vaginally, but seven of them had a history of cesarean section. For all subjects, mean age was 59.2 ± 12.7 years (range: 28–88 years), and 31.5% were 65 years or older. Fifty-six subjects (77%) were menopausal. The mean BMI was 25.0 ± 3.7 kg/m^2^. The average number of births was 3.1 ± 1.3, and 68% of patients had parity greater than 2. Thirty-five (48%) had a history of gynecological surgery. In the MUI subgroup, 83% of patients had parity >2. Overall, fifty women completed this study, while nine women in SUI, three in UUI, and eleven in MUI withdrew from the rehabilitation program. Reasons for withdrawal included personal reasons, job-related reasons, and family reasons. No adverse events were noted.

The vaginal pressure, PFM maximum recruitment and control accuracy were compared between week 0 (baseline) and week 6 or week 12 after the 12-week comprehensive rehabilitation program (Table 2). Vaginal pressure in the total study population, SUI and MUI groups increased significantly at week 6 (*p* = 0.000, 0.000, and 0.015, respectively) and week 12 (*p* = 0.000, 0.000, and 0.002, respectively) after intervention as compared with baseline. In the UUI group, the increase in vaginal pressure was not significant at week 6 but was significant at week 12 (*p* = 0.003), indicating that PFM contraction was improved after treatment. The PFM maximum recruitment in the total study population, SUI, and UUI groups increased significantly at week 6 (*p* = 0.000, 0.006, and 0.017, respectively) and week 12 (*p* = 0.000, 0.002, and 0.001, respectively) after intervention, compared with baseline. However, PFM maximum recruitment did not increase significantly in the MUI group at week 6 and week 12 after intervention. The control accuracy in the total study population and SUI groups increased significantly at week 6 (*p* = 0.007 and 0.025, respectively) and week 12 (*p* = 0.000 and 0.000, respectively) after intervention, compared with baseline; while in the UUI and MUI groups, results were not significant at week 6, but were statistically significant at week 12 (*p* = 0.002 and 0.005, respectively).

**Figure 2:**
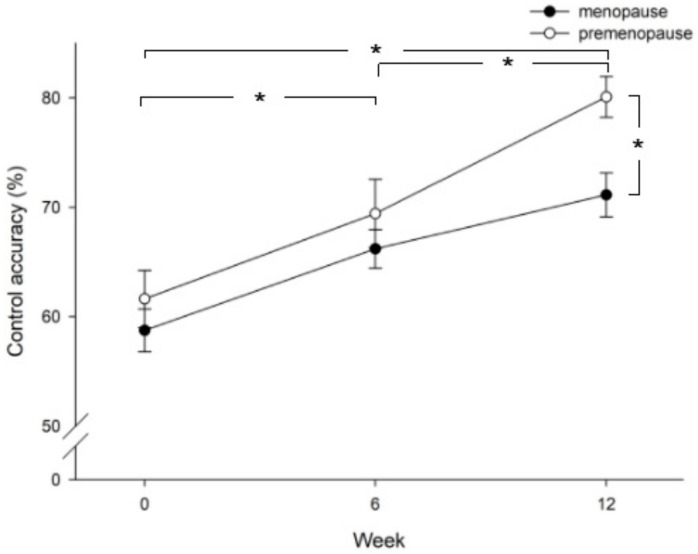
Comparison of control accuracy between patients in the pre-menopause and menopause stage at baseline (week 0), week 6, and week 12 after intervention. Significant differences are shown between menopause and pre-menopause groups (*p* = 0.013) and at week 12 vs. baseline (*p* = 0.000), week 6 vs. baseline (*p* = 0.036), and week 12 vs. week 6 (*p* = 0.002); *, *p* < 0.05.

**Figure 3:**
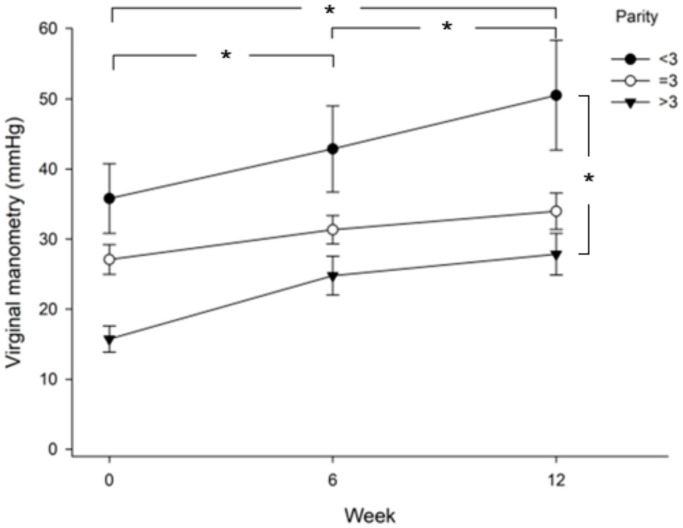
Comparison of vaginal pressure between groups of parity >3, =3, or <3. Significant differences are shown between the three groups at week 6 vs. baseline (*p* = 0.000), week 12 vs. baseline (*p* = 0.000), and week 12 vs. week 6 (*p* = 0.002). Vaginal pressure in the parity >3 group is significantly lower than that in the parity <3 group (least significant difference test, *p* = 0.003); *, *p* < 0.05.

Improvement in control accuracy was compared between patients in the menopause stage and the pre-menopause stage. Figure 2 shows that patients in the pre-menopause stage achieved higher control accuracy than patients in the menopause stage (*p* = 0.013), especially at week 12. In addition, both the menopause and pre-menopause groups showed significantly higher control accuracy at week 6 versus baseline (*p* = 0.036), at week 12 versus baseline (*p* = 0.000), and at week 12 versus week 6 (*p* = 0.002). When comparing vaginal pressure between groups of parity >3, =3, or <3, the results showed that patients with parity less than 3 achieved higher vaginal pressure than patients with parity equal to or more than 3 (Figure 3). The value was significantly higher in the parity < 3 group as compared with the parity >3 group (least significant difference test, *p* = 0.003). Those three groups showed significantly higher vaginal pressure at week 6 versus baseline (*p* = 0.000), at week 12 versus baseline (*p* = 0.000), and at week 12 versus week 6 (*p* = 0.002).

**Figure 4:**
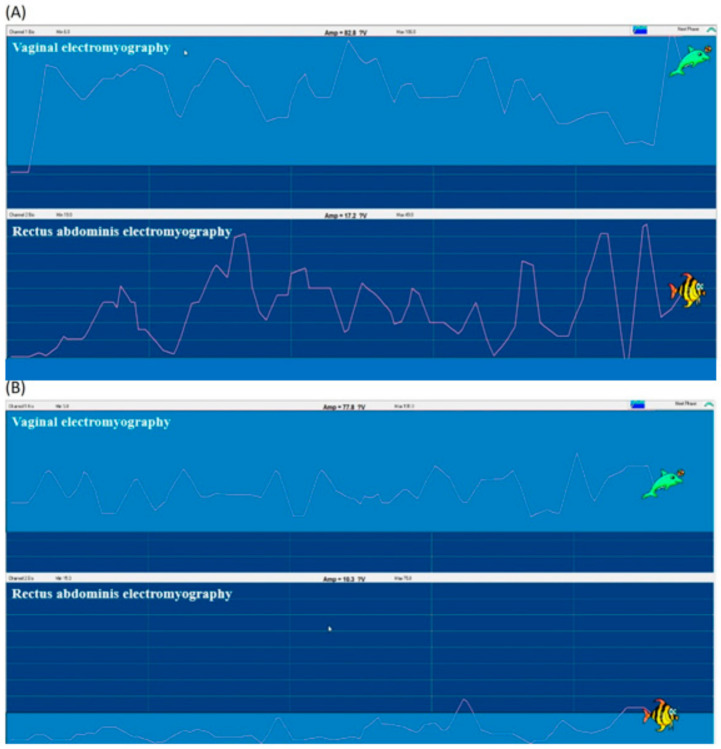
Vaginal electromyography and rectus abdominis electromyography examinations (**A**) before, and (**B**) after the rehabilitation program. (**A**) The rectus abdominis was inadequately induced for compensatory contraction. (**B**) After training, PFM contraction (measured with vaginal electromyography) was improved while the compensatory contraction of the rectus abdominis was remarkably reduced.

Vaginal electromyography and rectus abdominis electromyography examinations revealed that, before intervention, the rectus abdominis was inadequately used for compensatory contraction due to weakness of the PFM. After the intervention, vaginal electromyography indicated that PFM contracted correctly and effectively alone, indicating improved contraction. Compensatory contraction of the rectus abdominis was remarkably reduced (Figure 4).

**Figure 5:**
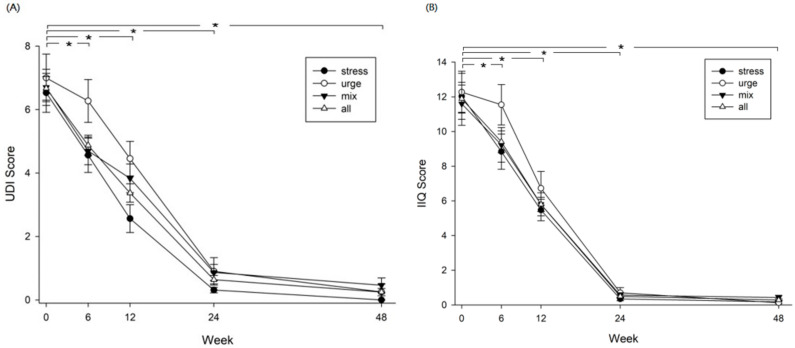
Urinary incontinence and impact on quality of life assessed with (**A**) the Short-form Urogenital Distress Inventory (UDI-6) and (**B**) the Short-form Incontinence Impact Questionnaire-7 (IIQ-7). Significant differences are shown in four groups at week 6 vs. baseline (*p* = 0.000), week 12 vs. baseline (*p* = 0.000), week 24 vs. baseline (*p* = 0.000), and week 48 vs. baseline (*p* = 0.000) in both (**A**,**B**); *, *p* < 0.

UDI-6 and IIQ-7 questionnaires were used to assess the urinary incontinence symptom distress and the impact on quality of life of patients after receiving the rehabilitation program. Changes in UDI-6 and IIQ-7 scores were followed continuously until week 48. For all groups of patients (SUI, UUI, and MUI), the UDI-6 and IIQ-7 scores both declined dramatically after rehabilitation. These scores decreased significantly at week 24, compared with baseline values and the effect was maintained to week 48, indicating significant improvements in urinary incontinence symptoms and quality of life (Figure 5, *p* = 0.000). In addition, results of the two scales revealed that the frequency of urine leakage episodes decreased significantly from 5.6 times per day before rehabilitation to 0.6 times per day after rehabilitation, indicating that the main problem of urinary incontinence was resolved to some extent. At the end, 58.9% (43/73) of total patients achieved complete dryness (no urinary leakage episode), of which 68.8% (22/32) in SUI, 63.6% (7/11) in UUI, and 46.7% (14/30) in MUI achieved complete dryness. Characteristics and types of urinary incontinence for the participants who achieved complete dryness after the intervention were analyzed with logistic regression analysis with forward stepwise variable selection method. Among the variables of age, BMI, menopause, total number of births, gynecological surgery, SUI, UUI, and MUI, only the total number of births (OR, 1.746; 95% CI, 1.087 to 2.804; *p* = 0.021) and gynecological surgery (OR, 0.314; 95% CI, 0.106 to 0.926; *p* = 0.036) were shown as significant predisposing factors for patients achieving dryness after rehabilitation (Table 3).

## 4. Discussion

In today’s aging society, urinary incontinence is a common symptom, especially in women after menopause [3,20]. However, despite the high prevalence of urinary incontinence, the rate of consultation is usually low, possibly due to lack of urgency, embarrassment, or other socio-cultural reasons [3]. Studies have pointed out that older age, having undergone gynecological surgery, menopause, obesity, and higher parity are risk factors for urinary incontinence [21,22]. In the present study, most subjects had at least one of these risk factors.

Previous studies have mainly focused on a single treatment modality or on a specific type of urinary incontinence. In the present study, a comprehensive rehabilitation program was designed and executed based on the actual status of each participating patient. In 2006, Capelini et al. [23] reported that 12 weeks of biofeedback and PFM training for patients with SUI significantly reduced the number of urine leakage episodes (from 8.14 per day to 2.57 per day), which is compatible with urine leakage results of the present study, which decreased from 5.6 times a day to 0.6 times a day. In 2014, a review article by Ghaderi et al. [24] on physical therapy for women with SUI mentioned that SUI was greatly improved in those receiving a supervised exercise program for at least three months. The effectiveness of the physical therapy was enhanced if the exercise program was based on intensity, duration, resembling functional task, and the position in which the PFM exercises were performed. For some women with SUI, biofeedback and electrical stimulation also appeared to be clinically useful and acceptable [24]. Results of the present study showed significant improvements in vaginal pressure at the 6th and 12th weeks of the comprehensive rehabilitation program as compared with baseline values. Radziminska et al. [25] systematically reviewed the impact of PFM training on the quality of life in women with urinary incontinence, finding that PFM training is an effective non-surgical intervention, especially for women with SUI. PFM training can significantly improve quality of life and is recommended as first-line conservative treatment for elderly women. The authors concluded that the PFM training should be provided under supervision and the duration should not be less than 6 weeks [25]. Our comprehensive rehabilitation program, similarly, is a 12-week intervention, resulting in significant improvements in PFM power and quality of life after intervention. In 2012, Fitz et al. [26] randomly divided 40 women with urinary incontinence into a biofeedback group and a control group and found that adding biofeedback to PFM training significantly improved PFM functions regarding power, endurance and fast (*p* < 0.001), referring increases in the reflex action of the fast muscle fibers and activation capacity of these muscles. Those authors suggested adding biofeedback to PFM training to reduce the symptoms of urinary incontinence and improve quality of life [26], as in the present study. In 2016, Ghaderi et al. [27] found that regular stabilization exercises focusing on PFM can increase PFM strength and endurance. In the present study, the exercise training focused mainly on PFM and core muscles. The UDI-6 scores and the IIQ-7 scores at weeks 24 and 48 after the training program still achieved significant improvements. This may be due to the subjects’ continued exercise training performed at home. One of the purposes of the present study was to encourage personalized exercise that participants can continue after the end of program to maintain the effect.

Although the present study evaluated patients with SUI, UUI, and MUI, results showed that the comprehensive program was particularly effective for subjects with SUI. In fact, the SUI subgroup achieved significant improvements in vaginal pressure, PFM maximum recruitment and control accuracy at the 6th and 12th weeks after treatment, while the other two subgroups did not achieve significant improvements in any of these three measures at the 6th week. The high efficacy of this comprehensive program in treating SUI may be due to PFM weakness accounting for the majority of cases with SUI, and that the main intention of this program is to induce, strengthen and control the strength of PFM.

The present study revealed that women with more than three births had poorer performance in vaginal pressure enhancement than women with less parity. Such poorer performance might be due to PFM dysfunction. It has been well established that higher parity is one of the risk factors for urinary incontinence, especially for women under 65 years of age and for the SUI type incontinence [28]. During delivery, when the fetal head turns downward and passes through the birth canal, it will exert pressure on the bladder and urethra, which may lead to strains of the pelvic floor muscle as well as damage to the nerve and blood vessels, resulting in the relaxation and atrophy of the entire pelvic muscle. For women with high parity (≥4), these strains and damage may accumulate after each childbirth, leading to PFM dysfunction.

The difference between the present study and previous studies is the increase in the training of PFM control. PFM plays a critical role in the neural control and function of detrusor [29,30]. The purpose of the newly designed training program was mainly to induce PFM activity, allowing patients to learn how to perform a correct PFM contraction. Continuous exercise stimulates the brain, nervous system and musculoskeletal system. In addition, via visual and auditory feedback, the best action strategy was found, a correct contraction pattern was established, and the patients corrected improper muscle usage patterns to increase PFM strength and endurance. Results showed that PFM control improved significantly after 12 weeks of training, suggesting that the comprehensive rehabilitation program effectively enhances PFM contraction, increases the accuracy of PFM control, ameliorates urinary incontinence symptoms, and improves the quality of life, especially for women with SUI. Efficacy was maintained up to 24 weeks and even 48 weeks after completion of the program, including that UDI-6 and IIQ-7 scores still improved significantly.

An additional advantage of the biofeedback device is that it increases fun for the patients during the training and treatment process through the customized game mode combined with audio-visual feedback, thereby improving patients’ motivation for treatment. The difficulty of the game can be adjusted and customized to accommodate patients’ abilities. Despite that the mean age of the included patients was around 60 years, they were able to understand and perform the training after it was explained. Results of this study suggest that the rehabilitation program is worth promoting in clinical practice.

One limitation of the present study is that the cohort size is small, only 50 patients completed the program. A survey by the Health Promotion Administration, Ministry of Health and Welfare, Taiwan, found that only 17.7% of women with urinary incontinence actually seek medical treatment [31]. In the present study, the small number of cases is likely to be due to the relatively conservative local folk customs. In addition, the use of the biofeedback device is a semi-invasive treatment method, so some of the study withdrawals may be due to patients’ inability to overcome psychological barriers. Another limitation is that our results were derived from patients from a single center. It was noted that all the included patients had normal weight; however, overweight or obesity is also among the risk factors that lead to SUI. Patients in the present study had normal weight, which may be due to the local population being dominated by farmers and labor, and the proportion of obese people is, therefore, relatively small. With the limited representativeness of the study population, findings cannot be generalized beyond the local community. Nevertheless, through the comprehensive rehabilitation program introduced in this study, patients with urinary incontinence can obtain individualized treatment and PFM training. Patients can learn to perform the PFM exercises correctly, and correct wrong actions via immediate feedback during the training process.

## 5. Conclusions

The comprehensive rehabilitation program effectively improves PFM contraction, increases the accuracy of PFM control, decreases urinary incontinence symptoms, and improves quality of life, especially for women with SUI and parity less than 3. Urinary incontinence can be well controlled, and quality of life can be improved even 48 weeks after training. Further promotion of health education related to urinary incontinence is needed within the community, providing the correct concepts and knowledge to improve motivation for medical treatment of urinary incontinence and improving compliance rates.

## Figures and Tables

**Table 1:** Demographic, Anthropometric, and Gynecologic Characteristics of included patients.

Group	Total (*n* = 73)	SUI (*n* = 32)	UUI (*n* = 11)	MUI (*n* = 30)
Age (years)	59.2 ± 12.7	55.0 ± 12.5	60.1 ± 9.9	63.2 ± 12.6
Menopause	56 (77%)	22 (69%)	9 (82%)	25 (83%)
Weight (kg)	60.8 ± 9.8	59.8 ± 7.7	59.6 ± 7.7	62.2 ± 12.1
Height (cm)	155.7 ± 5.5	155.7 ± 5.2	157.2 ± 6.8	155.2 ± 5.3
Body mass index (kg/m²)	25.0 ± 3.7	24.7 ± 3.0	24.1 ± 2.1	25.8 ± 4.6
Number of live births	3.1 ± 1.3	2.9 ± 1.0	2.5 ± 1.3	3.7 ± 1.4
Parity > 2	50 (68%)	22 (69%)	3 (27%)	25 (83%)
Gynecological surgery history	35 (48%)	13 (41%)	5 (46%)	17 (57%)

SUI, stress urinary incontinence; UUI, urgency urinary incontinence; and MUI, mixed urinary incontinence. Values are indicated in *n* (%) or mean ± standard deviation.

**Table 2:** Comparisons of measures between baseline vs. 6 weeks post-treatment and vs. 12 weeks post-treatment.

Group	Measurement	*n*	Week 0 ^a^ (Baseline)	Week 6 ^a^	Week 12 ^a^	*p*-Value	
						Week 6 vs. Baseline	Week 12 vs. Baseline
	Vaginal pressure (mmHg)	50	25.9 ± 19.4	32.9 ± 21.6	37.8 ± 22.6	0.000 *	0.000 *
Total	PFM maximum recruitment (uV)	43	26.8 ± 16.4	32.5 ± 16.9	37.6 ± 19.2	0.000 *	0.000 *
	Control accuracy (%)	44	58.8 ± 13.7	66.0 ± 12.5	73.2 ± 11.2	0.007 *	0.000 *
	Vaginal pressure (mmHg)	23	25.0 ± 13.1	31.7 ± 15.5	35.6 ± 17.1	0.000 *	0.000 *
SUI	PFM maximum recruitment (uV)	21	26.0 ± 14.9	32.2 ± 16.4	37.6 ± 20.5	0.006 *	0.002 *
	Control accuracy (%)	22	56.2 ± 15.4	65.6 ± 14.9	73.1 ± 9.1	0.025 *	0.000 *
	Vaginal pressure (mmHg)	8	37.2 ± 38.7	44.3 ± 43.6	51.4 ± 41.3	0.226	0.003 *
UUI	PFM maximum recruitment (uV)	8	26.5 ± 16.9	34.2 ± 15.6	38.4 ± 16.8	0.017 *	0.001 *
	Control accuracy (%)	8	62.9 ± 8.7	66.7 ± 12.5	78.7 ± 12.6	0.334	0.002 *
	Vaginal pressure (mmHg)	19	22.3 ± 12.6	29.4 ± 12.9	34.7 ± 16.4	0.015 *	0.002 *
MUI	PFM maximum recruitment (uV)	14	28.1 ± 19.1	32.0 ± 19.4	37.2 ± 19.8	0.270	0.062
	Control accuracy (%)	14	60.4 ± 13.4	66.3 ± 8.5	70.1 ± 13.1	0.249	0.005 *

^a^ Values represent mean ± standard deviation. SUI, stress urinary incontinence; UUI, urgency urinary incontinence; MUI, mixed urinary incontinence; and PFM, pelvic floor muscle. * Statistical significance: *p* < 0.05.

**Table 3:** Multivariate logistic regression analysis, with forward stepwise variable selection method.

Explanatory Variables	Odds Ratio ^a^	95% CI	*p*-Value
Total number of births	1.746	[1.087, 2.804]	0.021
Gynecological surgery	0.314	[0.106, 0.926]	0.036

^a^ Odds ratio for dryness; 95% CI: 95% confidence interval for the odds ratio.

## Data Availability

The data used to support the findings of this study are included within the article.
